# Alternate-day fasting differentially affects body composition, metabolic and immune response to fasting in male rats exposed to early-life adversity: Modulatory role of cafeteria diet

**DOI:** 10.1371/journal.pone.0313103

**Published:** 2025-03-03

**Authors:** Sara C. Sagae, Edson D. R. Paz, Bárbara Zanardini, Ana Claudia Amaral, Gabriela A. Bronczek, Patrícia Koehler-Santos, Jarbas R. de Oliveira, Celso R. Franci, Márcio V. F. Donadio, Parker J. Holman, Charlis Raineki

**Affiliations:** 1 Centro de Ciências Biológicas e da Saúde, Universidade Estadual do Oeste do Paraná, Cascavel, Brazil; 2 Departamento de Fisiologia Geral do Instituto de Biociências, Universidade de São Paulo, São Paulo, Brazil; 3 Departmento de Biologia Estrutural e Funcional, Instituto de Biologia, Universidade de Campinas, Campinas, Brazil; 4 Centro de Pesquisa Experimental, Hospital de Clínicas de Porto Alegre (HCPA), Porto Alegre, Brazil; 5 Laboratório de Biofísica Celular e Inflamação, Pontifícia Universidade Católica do Rio Grande do Sul (PUCRS), Porto Alegre, Brazil; 6 Departamento de Fisiologia, Faculdade de Medicina de Ribeirão Preto, Universidade de São Paulo, Ribeirão Preto, Brazil; 7 Departmento de Fisioterapia, Facultad de Medicina y Ciencias de la Salud, Universitat Internacional de Catalunya, Barcelona, Spain; 8 Department of Psychology, Brock University, St. Catharines, Ontario, Canada; Public Library of Science, UNITED KINGDOM OF GREAT BRITAIN AND NORTHERN IRELAND

## Abstract

The increased risk for obesity and metabolic disorders following early-life adversity is aggravated by poor diet (e.g., cafeteria diet). Alternate-day fasting (ADF) is a dietary regimen shown to improve immune and metabolic dysfunction related to obesity. Here, we evaluate if ADF can ameliorate the negative effects of early-life adversity and/or cafeteria diet on biological, immune and metabolic parameters. At weaning, animals reared under normal or adverse conditions (i.e., low bedding) were fed either standard chow or cafeteria diets *ad libitum* or subjected to an ADF regimen. In adulthood, we measured 24-hour fasted cholesterol, triglycerides, cytokines, oxidative stress markers, and body composition parameters including perigonadal, retroperitoneal, and brown fat pad weight. Animals exposed to early-life adversity respond differently to cafeteria diet and ADF. Adverse reared animals fed chow diet in the ADF regimen showed the largest reduction in body weight and perigonadal and retroperitoneal fat pad weight, the smallest increase in corticosterone levels, and the largest increase in TNF-α levels. However, the differential effects of the ADF regimen on body, perigonadal and retroperitoneal fat weight observed in adversely reared animals fed chow diet compared to controls were not present if the adversely reared animals were fed cafeteria diet in the ADF regimen. Furthermore, adversely reared animals fed cafeteria diet in the ADF regimen showed high IL-1β and IL-6 levels. Together, the data suggest that the altered vulnerability to metabolic and immune dysfunction following early-life adversity is not just due to the type of diet but also how the diet is consumed.

## Introduction

The clinical literature indicates that exposure to early-life adversity is associated with an increased lifetime risk for obesity and metabolic disorders, such as insulin resistance and type 2 diabetes [1–7]. Preclinical studies have corroborated these clinical findings, demonstrating in primate and rodent models that exposure to early-life adversity leads to increased rates of obesity and metabolic dysfunction [8–10]. We previously utilized a naturalistic rodent model of early-life adversity in which animals are reared in an environment with low bedding [for review see 11], and showed that this early-life adversity induces insulin resistance [12]. Importantly, insulin resistance was found in adversely reared animals fed standard chow. However, exposure to a more palatable diet—the cafeteria diet—uncovered and in some cases exacerbated this metabolic dysregulation. Indeed, adversely reared animals fed cafeteria diet had higher caloric intake, reduced pancreatic insulin secretion, more severe hepatic steatosis as well as increased plasma levels of IL-1𝛽 compared to normally reared animals fed the same diet [12]. Together, these data indicate that early-life adversity negatively programs the physiological systems regulating metabolic function and highlights the power that later dietary choices have in mediating the long-term effects of early-life adversity.

Although there has been progress in understanding how early-life adversity can increase risk for obesity and metabolic disorders, very few studies have evaluated possible interventions that may ameliorate the negative metabolic effects of early-life adversity [10,13,14]. Alternate-day fasting (ADF), a dietary regimen in which animals are exposed to multiple cycles alternating daily between 24-hour of fasting or 24-hour of *ad libitum* food access, has been shown to improve obesity-related metabolic dysfunction [15–18]. Nevertheless, only two studies have evaluated the effects of ADF following early-life adversity. The first study investigated maternally separated animals exposed to ADF during adolescence, finding that ADF resulted in more weight gain on the feed days, likely due to increased food intake, as well as more weight loss on fasted days when compared to controls [10]. Moreover, the altered food consumption observed in maternally separated animals exposed to ADF occurred in the context of increased responsiveness of the hypothalamic-pituitary-adrenal axis [10]. The second study showed that ADF rescued depressive-like behavior and increased dopaminergic activity in the ventral tegmental area/substantia nigra, nucleus accumbens, and hippocampus in maternally separated animals [14]. The results from these two studies indicate that animals exposed to early-life adversity show a differential response to ADF compared to *ad libitum* controls, suggesting the neurobiological processes regulating body weight and food intake are altered following early-life adversity.

Given the consistent evidence from clinical and preclinical literature that ADF improves overall health [16,17] and the limited evaluation of ADF’s effects in the context of early-life adversity, here we explore the effects of ADF on adversity-related metabolic dysfunction. To achieve this, we combined a standard ADF model with a well-established naturalistic low bedding model of early-life adversity to assess biological, metabolic, hormonal, and immune parameters. Additionally, given that later dietary choices can exacerbate the effects of early-life adversity [12,19–21], we tested the effects of ADF in adversely reared animals fed either standard lab chow (chow diet) or the high-salt, high-fat, low-fiber, energy dense cafeteria diet. We hypothesize that exposure to early-life adversity will interact with the negative effects of cafeteria diet consumption; moreover, animals exposed to early-life adversity will differentially respond to ADF regardless of diet.

## Methods

### Animals

Pregnant Wistar rats were obtained from the colony of Universidade Estadual do Oeste do Paraná and maintained at a constant temperature (22 ±  1°C) and a 12-hour light-dark cycle (lights on at 7 am) with *ad libitum* access to water and standard rodent chow (Algomix, Brazil). Approximately 7 days before delivery, females were housed individually (41 cm long X 34 cm wide X 17 cm high) and the presence of pups was checked twice daily. On the day of birth (postnatal day 0; PN0), litters were culled to 8 pups with an attempt to preserve an equal number of males and females per litter. To control for litter effects, no more than 2 animals per litter were used in each experimental group. All experiments were approved by the Universidade Estadual do Oeste do Paraná Animal Care Committee (protocol # 39/17) and were performed in accordance with National Institutes of Health (NIH) Guide for the Care and Use of Laboratory Animals and the Canadian Council on Animal Care guidelines.

### Naturalistic early-life adversity paradigm

**Fig 1:**
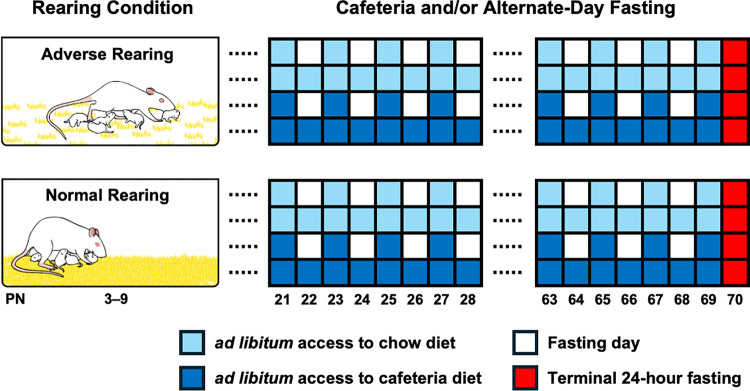
Schematic representation of the experimental design. From PN3-9, litters were randomly exposed to normal or adverse rearing conditions (i.e., low bedding). After weaning (PN21), male offspring were exposed to one of two experimental diets: chow or cafeteria. Diets were administered using two different feeding regimens: *ad libitum* (AL), where animals received *ad libitum* access to food (chow or cafeteria diet); and alternate-day fasting (ADF), where animals were exposed to repeated 24-hour fasting followed by 24-hour refeeding cycles during which they had *ad libitum* access to chow or cafeteria diet. At PN70, after 24 hours of fasting, animals were terminated to measure perigonadal, retroperitoneal, and brown fat pad weight and to assess cholesterol, triglycerides, corticosterone, cytokines, and oxidative stress markers.

At PN3, litters were randomly assigned to either adverse or normal rearing conditions (Fig 1). For the adverse rearing litters, mothers were housed from PN3-9 with limited nesting/bedding material (1000mL, 1.2 cm layer). This limited bedding environment has been shown to decrease dams’ ability to construct nests and results in an abusive-like maternal behavior phenotype [for review see 11]. Indeed, mothers provided with limited bedding material spend more time away from pups and show more rough handling (i.e., aggressively grooming pups, transporting pups by limb, step/jump on pups) [22,23]. For the normal rearing litters, mothers were housed from PN3-9 in cages with abundant bedding material (4500mL, 5 cm layer) for nest building, during which time they were not disturbed.

### Cafeteria diet

Beginning at weaning (PN21), male rats from both normal and adverse rearing groups were pair-housed and randomly divided into two dietary experimental groups: 1) Chow diet; fed standard rodent chow (Table 1; Algomix, Brazil; 2.95 kcal/g) and water *ad libitum*; and 2) Cafeteria diet; palatable food items comprising salami, ham, sausages, snacks, cakes, breads, cookies, and soft drinks (alternated daily between Pepsi™ and Guaraná Antarctica™) as detailed in Table 1. Those were offered together with a cafeteria pellet diet (Table 2) and water *ad libitum* [12,24,25]. The cafeteria pellet diet was made every other day by combining melted chocolate (25 g), powdered standard lab chow (37.5 g), powdered Biscuit Maizena (12.5 g), and toasted peanuts (25 g). The mixture was baked for 40 min at 180 °C and, once cooled, cut in square pellets of 4 x 4 cm. The cafeteria diet was offered from PN21 until the experimental day (PN70 ±  1; adulthood).

**Table 1:** Nutritional details of the cafeteria and standard chow diets.

	Qtd (g or ml*)	Kcal	Carbohydrates (g)	Protein (g)	Fat (g)	Na (mg)
Chow (Algomix, Brazil)	1	2.95	0.55	0.22	0.04	2.70
Salami (Majestade, Brazil)	1	3.38	0.00	0.32	0.24	12.48
Bread (Seven Boys, Brazil)	1	2.95	0.53	0.09	0.04	4.70
Snack Yokitos (Yoki, Brazil)	1	4.80	0.60	0.06	0.24	11.04
Smoked sausage (Perdigão, Brazil)	1	3.18	0.01	0.18	0.32	15.73
Chocolate cake (Nutrella, Brazil)	1	3.25	0.50	0.05	0.12	6.18
Biscuit Maizena (Nestlé, Brazil)	1	4.33	0.70	0.10	0.12	3.63
Ham (Sadia, Brazil)	1	1.55	0.00	0.17	0.09	8.33
Snack Fritello (Pavioli, Brazil)	1	5.08	0.52	0.08	0.29	6.40
Peanuts	1	5.20	0.16	0.26	0.49	0.18
Pepsi (PepsiCo, Brasil)^*^	1	0.40	0.11	0.00	0.00	0.05
Guaraná Antartica (AmBev, Brasil)^*^	1	0.40	0.11	0.00	0.00	0.05

**Table 2:** Nutritional details of the cafeteria pellet diet.

	Qtd (g)	Kcal	Carbohydrates (g)	Protein (g)	Fat (g)	Na (mg)
Chow (Algomix, Brazil)	37.5	110.6	20.6	8.2	1.5	75.0
Chocolate (Garoto, Brazil)	25.0	127.0	13.0	2.0	7.2	160.0
Biscuit Maizena (Nestlé, Brazil)	25.0	130.0	14.0	1.5	8.0	12.5
Peanuts	12.5	53.6	9.1	1.7	1.5	54.1
**Total**	100	421.2	56.7	13.4	18.2	301.6

### Alternate-day fasting (ADF)

Besides dividing the normal and adverse rearing groups into chow or cafeteria diet, at PN21 animals were also randomly subdivided two different feeding regimens: 1) *Ad libitum* (AL); animals received *ad libitum* access to food (chow or cafeteria diet) and water for the whole experimental period; and 2) Alternate-day fasting (ADF); animals were exposed to repeated 24-hour fasting followed by a 24-hour refeeding cycles 2 hours after lights on (9 am). During the 24-hour fasting period, animals were deprived of food but had *ad libitum* water; during the 24-hour refeeding period, animals had *ad libitum* access to chow or cafeteria diet depending on their dietary experimental groups assignment and water (Fig 1).

### Experimental design

Normally and adversely reared animals were provided access to one of two experimental diets: chow or cafeteria (Fig 1). Diets were administered using two different feeding regimens, AL and ADF, resulting in 8 experimental groups: 1) normally reared, chow diet, AL; 2) normally reared, chow diet, ADF; 3) normally reared, cafeteria diet, AL; 4) normally reared, cafeteria diet, ADF; 5) adverse reared, chow diet, AL; 6) adverse reared, chow diet, ADF; 7) adverse reared, cafeteria diet, AL; and 8) adverse reared, cafeteria diet, ADF. For the specific number of animals in each experimental group and outcome, see S1 Table in S1 File.

At PN70, after 24 hours of food restriction (i.e., terminal 24-hour fasting), animals were weighed and decapitated to collect blood in heparinized tubes for assays of cholesterol, triglycerides, corticosterone, cytokines, and oxidative stress markers and to measure perigonadal, retroperitoneal, and brown fat pad weight. Decapitation was performed without prior anesthesia by a certified and trained experimenter given that several of our experimental outcomes are sensitive to anesthesia [26]. To evaluate obesity incidence, the ratio of perigonadal, retroperitoneal, and brown fat (g)/ 100 g of body weight was used [12,24]. All assays were conducted without knowledge of the experimental conditions.

### Cholesterol and triglycerides

Plasma cholesterol and triglycerides were measured using commercially available kits from Labtest Diagnostica (Minas Gerais, Brazil) and run according to the manufacturer’s instructions. The lower limit of detection (LLOD) for cholesterol was 2 mg/dL, and the intra- and inter-assay coefficients of variation were 1.2% and 2.2%, respectively. The LLOD for triglycerides was 3 mg/dL, and the intra- and inter-assay coefficients of variation were 1.3% and 1.9%, respectively.

### Corticosterone

Plasma corticosterone was measured by ^3^H-labeled hormone (Amersham, Piscataway, NJ, USA) radioimmunoassay, with specific antibody (cat# C8784) at 1:10,000 dilution and standards (cat# C2505) from Sigma Inc. (USA). Separation of the free and bound fractions was performed with charcoal-dextran (0.5/0.05%). Prior to radioimmunoassay, corticosterone was extracted from plasma using ethanol, with all samples processed in the same assay. The LLOD and the coefficient of intra-assay variation were 0.04 ng/ml and 4.5%, respectively.

### Cytokines

In plasma, multiple soluble cytokines (TNF-α, IL-1β, IL-10, and IL-6) were simultaneously measured using a Luminex Multiplex Assay kit (Thermo Fisher Scientific) using a luminometer Luminex^®^ 100/200 (Luminex Corporation, Austin, TX) and the results were analyzed using the software xPONENT^®^ Solutions software (Luminex Corporation). The LLOD for each cytokine was: TNF-α: 0.4 pg/mL, IL-1β: 1.1 pg/mL, IL-10: 1.6 pg/mL, and IL-6: 1.9 pg/mL. The intra- and inter-assay coefficients of variation were both < 10%.

### Oxidative stress

Thiobarbiture acid reactive substances (TBARS), an index of lipid peroxidation, were measured in plasma samples [12,27]. Samples (125 µL) were incubated using 375 µL of 0.67% thiobarbituric acid (TBA) and 250 µL of 10% trichloroacetic acid (TCA). The mixture was vortexed, and the reaction carried out in a boiling water bath for 1 h. After ice cooling, 1.5 mL of butyl alcohol was added to the mixture. The resulting pink-stained TBARS were determined spectrophotometrically at 535 nm. The method’s sensitivity is 1 µM.

### Statistical analyses

All data are expressed as mean ±  SEM and were analyzed by three-way ANOVA (rearing condition, diet and feeding regimen as factors). When significant, ANOVAs were followed by Newman-Keuls *post hoc* tests. Further analyses utilized planned pairwise comparisons to test the *a priori* hypotheses that: 1) exposure to adverse rearing will alter metabolic, hormonal, and immune function compared to that observed in normally reared animals; 2) cafeteria diet will alter metabolic, hormonal, and immune function compared to that observed in animals consuming the chow diet, and 3) ADF will alter metabolic, hormonal, and immune function compared to that observed in animals in the AL regimen. Outliers were identified and removed using the Robust regression and Outlier removal (ROUT) method with Q = 0.05. In all cases, differences were considered significant when p ≤  0.05. For all tests, the software packages Statistica 13 (Statsoft, USA) and GraphPad Prism 10.0 (USA) were used. All statistical results (main effects and interactions) can be found in S2–S4 Tables in S1 File. Only the significant main effects and interactions are reported in the results section.

## Results

### Body composition

**Fig 2:**
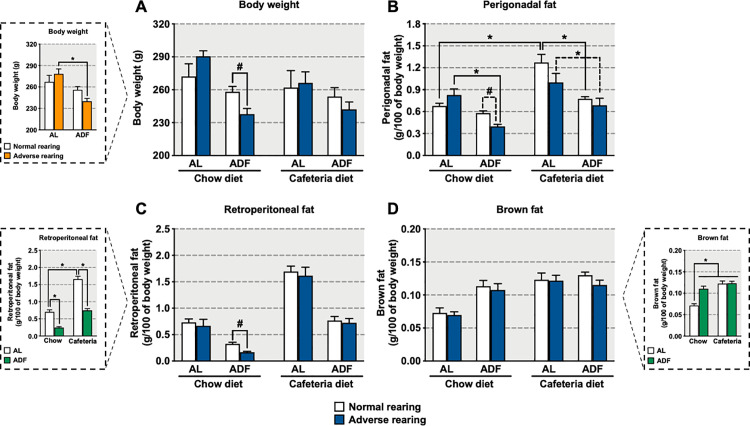
Effects of early-life adversity on (A) body weight, (B) perigonadal fat, (C) retroperitoneal fat, and (D) brown fat in animals fed cafeteria diet in an alternate-day fasting (ADF) regimen. Bars represent mean +  SEM (n =  4-9 for all groups). *  indicates significant differences based on the *post-hoc* tests for interactions or main effects; # indicates significant differences based on *a priori* comparison. Box for graph A shows the statistical results for the interaction between rearing condition and feeding regimen, regardless of diet. Boxes for graphs C and D show the statistical results for the interaction between diet and feeding regimen, regardless of rearing condition. For *  or #, **p** ≤  0.05. AL: *ad libitum*.

Consumption of cafeteria diet from PN21 to PN70 did not result in increased body weight regardless of rearing condition or feeding regimen (Fig 2A). However, in adversely reared animals, the ADF feeding regimen reduced body weight as compared to AL-regimented controls, regardless of diet consumed (Fig 2A box) (significant main effect of feeding regimen [F_(1,34)_ =  15.46, p =  0.0003], and significant interaction between rearing condition and feeding regimen [F_(1,34)_ =  4.70, p =  0.04]). Moreover, when fed a chow diet using ADF feeding regimen, adversely reared animals showed reduced body weight relative to their normally reared counterparts [*a priori* analysis for body weight comparing normally and adversely reared animals fed chow diet on the ADF regimen (p =  0.02)].

Among chow fed AL-regimented animals, adverse rearing did not affect perigonadal fat pad weight (Fig 2B). When offered cafeteria diet, normally reared, AL-regimented animals showed the largest increases in perigonadal fat pad weight; conversely, fat pad weights from adversely reared animals fed chow were not different from adversely reared animals fed cafeteria diet. Importantly, when consuming chow diet, the ADF regimen reduced perigonadal fat pad weight in adversely but not normally reared animals; when consuming cafeteria diet, the ADF regimen reduced perigonadal fat pad weights in both normally and adversely reared animals. Finally, *a priori* analysis revealed adversely reared animals consuming chow diet were more sensitive to the ADF regimen, as they showed reduced fat pad weight compared to their normally reared counterparts (significant main effects of diet [F_(1,33)_ =  37.03, p <  0.0001] and feeding regimen [F_(1,33)_ =  42.41, p <  0.0001], and significant interaction among rearing condition, diet and feeding regimen [F_(1,33)_ =  6.29, p =  0.02]; *a priori* analysis for perigonadal fat pad comparing normally and adversely reared animals fed chow diet on the ADF regimen [p =  0.002]).

Animals consuming cafeteria diet on an AL regimen exhibited increased retroperitoneal fat pad weights compared to animals consuming chow diet on an AL regimen, independent of rearing condition (Fig 2C, 2C box). Additionally, the ADF regimen reduced retroperitoneal fat pad weights in animals consuming chow and cafeteria diet, regardless of rearing condition. However, adversely reared animals consuming chow diet on an ADF regimen had significantly smaller retroperitoneal fat pad weights than their normally reared counterparts (significant main effects of diet [F_(1,34)_ =  157.55, p <  0.0001] and feeding regimen [F_(1,34)_ =  138.05, p <  0.0001], and significant interaction between diet and feeding regimen [F_(1,34)_ =  15.53, p =  0.0004]; *a priori* analysis for retroperitoneal fat pad comparing normally and adversely reared animals fed chow diet on the ADF regimen [p =  0.02]).

All animals consuming cafeteria diet, regardless of regimen, as well as animals consuming chow diet on the ADF regimen, exhibited increased brown fat pad weights compared to animals consuming chow diet on the AL regimen, independent of rearing condition (Fig 2D, 2D box; significant main effects of diet [F_(1,46)_ =  32.67, p <  0.0001] and feeding regimen [F_(1,46)_ =  12.62, p =  0.0009], and significant interaction between diet and feeding regimen [F_(1,46)_ =  12.37, p =  0.0009]).

### Metabolic and hormonal parameters

**Fig 3:**
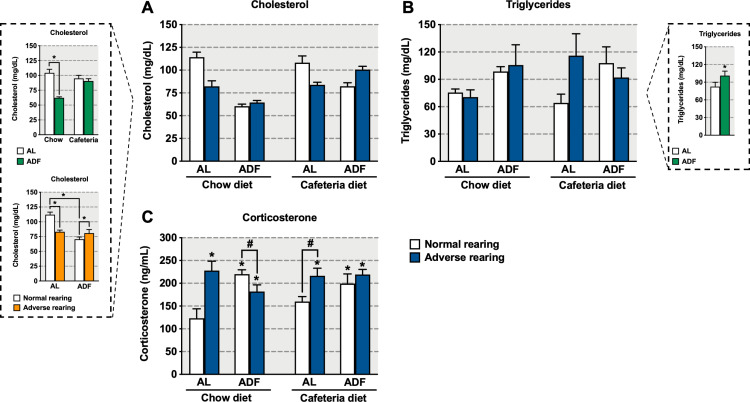
Effects of early-life adversity on (A) cholesterol, (B) triglycerides, and (C) corticosterone levels in animals fed cafeteria diet in an alternate-day fasting (ADF) regimen. Bars represent mean +  SEM (n =  4-9 for all groups). *  indicates significant differences based on the *post-hoc* tests for interactions or main effects; # indicates significant differences based on *a priori* comparison. Box for graph A shows the statistical results for the interaction between rearing condition and feeding regimen, regardless of diet and the statistical results for the interaction between diet and feeding regimen, regardless of rearing condition. Box for graph B shows the statistical results for the main effect of feeding regimen, regardless of rearing condition and diet. For *  or #, **p** ≤  0.05. AL: *ad libitum*.

Normally reared animals exposed to the ADF regimen showed reduced terminal 24-hour fasting cholesterol levels compared to animals exposed to the AL regimen, regardless of diet consumed (Fig 3A, 3A box). Furthermore, adversely reared animals on the AL regimen had lower cholesterol levels than their normally reared, AL-regimented counterparts, regardless of diet. Importantly, adversely reared animals on the ADF regimen, regardless of diet, exhibited increased cholesterol levels as compared to their normally reared counterparts (significant main effects of rearing condition [F_(1,40)_ =  6.59, p =  0.01], diet [F_(1,40)_ =  16.36, p =  0.0002] and feeding regimen [F_(1,40)_ =  37.09, p <  0.0001]; significant interaction between rearing condition and feeding regimen [F_(1,40)_ =  35.30, p <  0.0001]). We also detected a significant interaction between diet and feeding regimen, such that exposure to the ADF regimen reduced cholesterol levels in animals exposed to chow diet regardless of rearing condition (Fig 3A box; significant interaction between diet and feeding regimen [F_(1,40)_ =  22.24, p <  0.0001]).

Exposure to the ADF regimen increased terminal 24-hour fasting triglycerides levels independent of rearing condition and diet (Fig 3B, 3B box; significant main effect of feeding regimen [F_(1,40)_ =  4.44, p =  0.04]).

Terminal 24-hour fasting corticosterone levels were increased in adversely reared animals consuming either chow or cafeteria diet on the AL regimen (Fig 3C). Moreover, all ADF-regimented animals showed increased terminal 24-hour fasting corticosterone levels compared to normally reared animals consuming chow diet on the AL regimen. However, among normally reared, AL-regimented animals, consuming cafeteria diet did not alter corticosterone levels compared to animals consuming chow diet. Moreover, among ADF-regimented animals consuming chow diet, adversely reared animals exhibited lower corticosterone levels compared to their normally reared animals counterparts. Conversely, among AL-regimented animals consuming cafeteria diet, adversely reared animals exhibited higher terminal 24-hour fasting corticosterone levels as compared to their normally reared counterparts (significant main effect of rearing condition [F_(1,38)_ =  9.03, p =  0.005], significant interaction between rearing condition and feeding regimen [F_(1,38)_ =  14.23, p =  0.0005], and significant interaction among rearing condition, diet and feeding regimen [F_(1,38)_ =  4.99, p =  0.03]; *a priori* analysis for corticosterone levels comparing normally and adversely reared animals fed chow diet on the ADF regimen [p =  0.05]; *a priori* analysis for corticosterone levels comparing normally and adversely reared animals fed cafeteria diet on the AL regimen [p =  0.02]).

### Cytokines and oxidative markers

**Fig 4:**
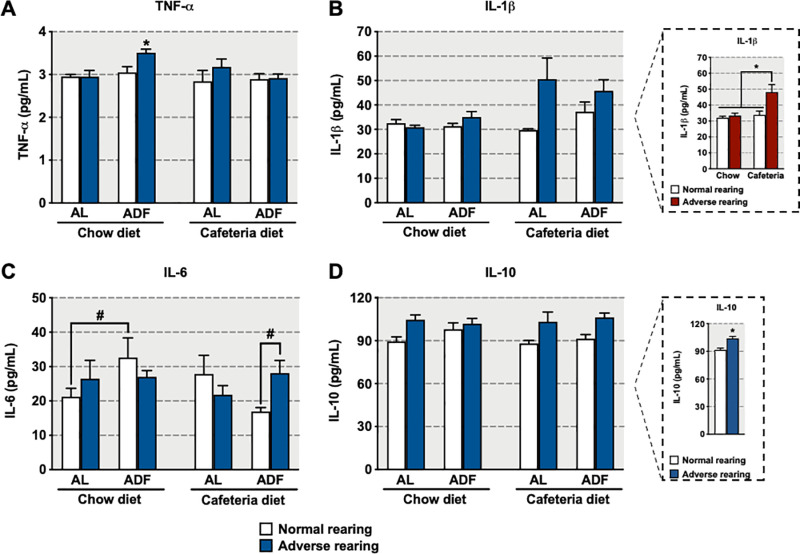
Effects of early-life adversity on (A) TNF-α, (B) IL-1β, (C) IL-6, and (D) IL-10 levels in animals fed cafeteria diet in an alternate-day fasting (ADF) regimen. Bars represent mean +  SEM (n =  4-9 for all groups). *  indicates significant differences based on the *post-hoc* tests for interactions or main effects; # indicates significant differences based on *a priori* comparison. Box for graph B shows the statistical results for the interaction between rearing condition and diet regardless of feeding regimen. Box for graph D shows the statistical results for the main effect of rearing condition, regardless of feeding regimen and diet. For *  or #, **p** ≤  0.05. AL: *ad libitum*.

Adversely reared animals consuming either chow or cafeteria diet on the AL regimen did not show altered terminal 24-hour fasting TNF-α levels (Fig 4A), though adversely reared animals consuming chow diet on the ADF regimen showed higher TNF-α levels than their normally reared counterparts (significant main effect of rearing condition [F_(1,40)_ =  4.61, p =  0.04], significant interaction between diet and feeding regimen [F_(1,40)_ =  5.09, p =  0.03], and significant interaction among rearing condition, diet and feeding regimen [F_(1,40)_ =  4.15, p =  0.05]).

Regardless of feeding regimen, adversely reared animals consuming cafeteria diet showed increased terminal 24-hour fasting IL-1β levels compared to all normally reared animals independent of diet as well as adversely reared animals consuming chow diet (Fig 4B, 4B box; significant main effects of rearing condition [F_(1,40)_ =  7.73, p =  0.008] and diet [F_(1,40)_ =  8.76, p =  0.005], and significant interaction between rearing condition and diet [F_(1,40)_ =  5.75, p =  0.02]).

Adversely reared animals consuming either chow or cafeteria diet on the AL regimen did not show altered terminal 24-hour fasting IL-6 levels (Fig 4C). However, in normally reared animals consuming chow diet, exposure to the ADF regimen increased IL-6 levels. Among animals consuming cafeteria diet on the ADF regimen, adversely reared animals had higher terminal 24-hour fasting IL-6 levels as compared to normally reared animals (significant interaction among rearing condition, diet and feeding regimen [F_(1,40)_ =  7.07, p =  0.01], *a priori* analysis for IL-6 comparing normally reared animals consuming chow diet and AL regimen with normally reared animals consuming chow diet and ADF regimen [p =  0.05]; *a priori* analysis for IL-6 comparing normally and adversely reared animals consuming cafeteria diet on the ADF regimen [p =  0.02]).

Adversely reared animals showed increased terminal 24-hour fasting IL-10 levels compared to normally reared animals, regardless of diet or feeding regimen (Fig 4D, 4D box; significant main effect of rearing condition [F_(1,40)_ =  17.88, p <  0.0001]).

**Fig 5:**
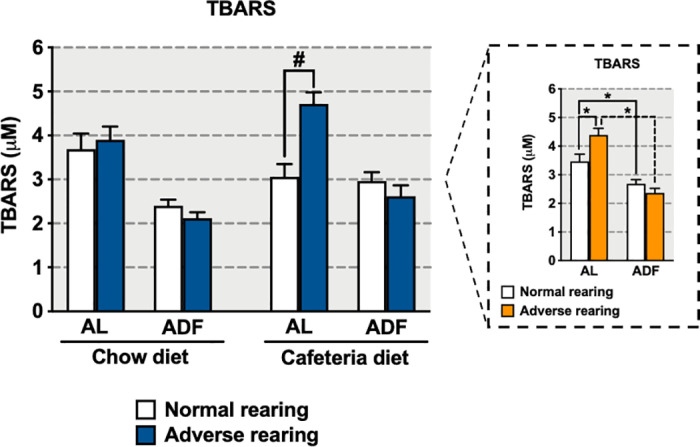
Effects of early-life adversity on TBARS levels in animals fed cafeteria diet in an alternate-day fasting (ADF) regimen. Bars represent mean +  SEM (n =  4-9 for all groups). *  indicates significant differences based on the *post-hoc* tests for interactions or main effects; # indicates significant differences based on *a priori* comparison. Box shows the statistical results for the interaction between rearing condition and feeding regimen, regardless of diet. For *  or #, **p** ≤  0.05. AL: *ad libitum*.

Adversely reared animals exposed to the AL regimen showed increased terminal 24-hour fasting TBARS levels independent of diet (Fig 5, 5 box), with further *a priori* analysis indicating this effect was driven by animals consuming cafeteria diet (e.g., only adversely reared animals consuming cafeteria diet on the AL regimen were different form normally reared animals). Moreover, exposure to the ADF regimen reduced terminal 24-hour fasting TBARS levels in both normally and adversely reared animals regardless of diet (significant main effect of feeding regimen [F_(1,40)_ =  44.43, p <  0.0001], and significant interaction between rearing condition and feeding regimen [F_(1,40)_ =  10.02, p =  0.003]).

## Discussion

The clinical and preclinical literature have demonstrated that consumption of high-calorie, sugar/fat-enriched foods may be more harmful for individuals exposed to early-life adversity [1–10,12]. The present study expands on these findings by showing that the altered vulnerability to obesity and metabolic dysfunction faced by individuals exposed early-life adversity is not just due to the type of diet, but also how the diet is consumed. Indeed, our present results demonstrate that the effects of ADF are augmented in adversely reared animals when fed a chow diet, but not when fed a cafeteria diet. This exaggerated effect of ADF in adversely reared animals is apparent due to a larger reduction in perigonadal fat, retroperitoneal fat, and body weight when fed a chow diet in the ADF regimen, but not when fed a cafeteria diet in the ADF regimen. Additionally, we observed a reduction in corticosterone and an increase in TNF-α only when adversely reared animals were fed a chow diet in the ADF regimen, but not when fed a cafeteria diet in the ADF regimen.

### Effects of cafeteria diet and ADF on body composition in animals exposed to early-life adversity

The current data replicate the literature [12,24,25,28,29] demonstrating that exposure to cafeteria diet on an AL regimen results in increased perigonadal and retroperitoneal fat pad weight, suggesting that exposure to cafeteria diet leads to obesity. Importantly, the cafeteria diet-induced effect was independent of early-life experiences, as both normally and adversely reared animals showed increased perigonadal and retroperitoneal fat pad weight. However, despite this fat increase, overall body weight was not affected by the cafeteria diet on the AL regimen. This result contrasts with the majority of studies, including ours, where consumption of cafeteria diet typically results in a significant increase in body weight [12,30]. However, there are reports in the literature where cafeteria diet did not lead to increased body weight [31–36]. Various factors such as age, specific type of food offered, and duration of diet exposure may contribute to the different effects on body weight. Notably, the age diet exposure starts has been shown to affect body weight gain [32,37]. Indeed, a six-week exposure to cafeteria diet resulted in increased body weight in adult animals, but not in adolescent animals [37], presumably due to the increased energy demand needed to support the adolescent growth spurt. Nevertheless, both age groups fed cafeteria diet showed an increase in fat deposition. A similar lack of increased body weight with increased fat deposition was also observed when young animals were exposed to a high-energy diet for five weeks [38]. Moreover, the discrepancy between the lack of body weight increase in the current study and the increased body weight observed in our previous study [12] might be related to differences in the exposure period. In the current study, animals were exposed to a cafeteria diet for seven weeks (P21-P70), whereas in our previous study, exposure lasted eleven weeks (P21-P98). It is therefore likely that the age at which the cafeteria diet started and the relatively shorter exposure period in the current study contributed to the lack of effects on body weight.

ADF, which involves a fasting day (i.e., where no food is consumed) alternated with a feeding day (i.e., where food is consumed *ad libitum*), has been shown to reduce total and visceral fat mass and improve metabolic parameters in humans [17,39,40]. Our results indicate that normally reared animals consuming chow diet in the ADF regimen show reduced retroperitoneal fat accumulation, suggesting that, similar to other studies in humans and other animal models [15,41–43], our ADF protocol was effective in preventing fat deposition. Importantly, this ADF effect was even more pronounced in adversely reared animals, which showed a larger reduction of perigonadal and retroperitoneal fat accumulation in the context of reduced body weight. The exacerbated effect of ADF observed in adversely reared animals highlights the fact that dietary interventions with the goal of improving obesity and related metabolic dysfunction may have differential effects depending on previous early-life experiences.

Animals consuming cafeteria diet on the ADF regimen also showed reduced perigonadal and retroperitoneal fat accumulation as compared to AL animals, regardless of rearing condition. Indeed, in contrast to our findings in chow-fed animals, we did not observe any additional reduction in adversely versus normally reared animals; in other words, the reduction in fat accumulation was greater only in adversely reared animals fed chow diet as compared to their cafeteria fed counterparts. Indeed, this reduction of fat pad accumulation in adversely reared animals on the ADF regimen was absent in cafeteria-fed animals, as both normally and adversely reared animals exposed to the ADF regimen show similar reductions in perigonadal and retroperitoneal fat accumulation. These results suggest that consumption of cafeteria diet by adversely reared animals eliminates the differential effects of the ADF regimen. Finally, fat pad weights in animals fed cafeteria diet on the ADF regimen were similar to animals fed chow diet in AL regimen regardless of rearing condition, suggesting that the ADF feeding regimen attenuated some of the effects of consuming cafeteria. The more effective reduction of perigonadal and retroperitoneal fats induced by ADF in adversely reared animals fed chow diet as compared to cafeteria diet may be the result of the chow diet’s lower fat and carbohydrate levels. Indeed, fat storage becomes the largest energy supplier during fasting periods [17,40], often resulting in reductions of fat mass [39,44]. Thus, the less calorically dense formulation of chow diet may have facilitated the use of visceral adipose tissue in the absence of ingesting large amounts of energy.

Exposure to ADF increased brown fat weight of both normally and adversely reared animals, regardless of diet offered, replicating previous data in normally reared mice exposed to intermittent fasting [45]. Unlike visceral fat, brown fat has been associated with caloric expenditure for thermogenic activity [46]. Additionally, brown adipose tissue plays an important role in metabolic control, including reduced body fat, increased insulin sensitivity, and control of metabolic factors [47,48]. Brown fat also has been shown to protect against metabolic disorders in animals with diet-induced obesity [49]. All of these functions of brown fat may have contributed to the reduction in perigonadal and retroperitoneal fat pad weight induced by ADF in normally and adversely reared animals fed chow or cafeteria diet relative to their own AL counterparts. Interestingly, we observed that cafeteria diet also promoted an increase in brown fat accumulation in both normally and adversely reared animals regardless of feeding regimen. Brown adipose tissue activity increases following food intake, resulting in increased respiratory rate and mitochondrial activity as well as greater glucose utilization and increased fatty acid synthesis [50,51]. Exposure to cafeteria diet has previously been shown to further increase the activity of brown adipose tissue [52], which may have contributed to our observation of increased brown fat accumulation in our cafeteria consuming animals. However, our results of increased brown fat in both normally and adversely reared animals fed cafeteria diet indicates that early-life adversity may not differentially affect brown fat accumulation in response to high-calorie, sugar/fat-enriched foods such as those in the cafeteria diet.

### Metabolic and hormonal responses to cafeteria diet and ADF in animals exposed to early-life adversity

Exposure to the ADF regimen reduced terminal 24-hour fasting cholesterol levels in normally reared animals regardless of diet, replicating previous findings in normally reared mice exposed to ADF and high-fat diet [53]. However, the beneficial effects of ADF were not as robust in adversely reared animals, as they showed higher cholesterol levels compared to normally reared, ADF-regimented animals irrespective of diet consumed. Nevertheless, our present data demonstrate that exposure to early-life adversity led to reduced terminal 24-hour fasting cholesterol levels, an effect that was observed in adversely reared animals fed chow and cafeteria diet on the AL regimen, replicating some of our previous findings in *ad libitum* fed animals [12]. Together, these results indicate that early-life adversity alters the metabolic pathways regulating cholesterol production and suggests that dietary interventions, such as ADF, may have differential effects in individuals exposed to early-life adversity.

Previous studies indicate that rodents exposed to intermittent fasting using standard rodent chow show a decrease [41,54] or no change [53] in plasma triglycerides levels. Our results indicate that exposure to the ADF regimen increased triglycerides levels in normally and adversely reared animals consuming chow and cafeteria diet. Of note, a key difference between these previous results and the current data is that we measured fasting triglyceride levels while other studies have measured triglycerides after a refeeding period. ADF protected normally and adversely reared animals from the expected decrease in triglyceride levels following fasting regardless of diet [55], suggesting that ADF induced lipolysis, resulting in triglyceride level elevation for muscle utilization that favors body weight reductions and glucose conservation.

In the current study, terminal 24-hour fasting in adversely reared animals, whether fed a chow or cafeteria diet on the AL regimen, resulted in a higher increase in corticosterone levels compared to normally reared animals under the same conditions, suggesting that adversely reared animals are hyperresponsive to stressors such as food deprivation. A similar profile has been shown in maternally separated rats exposed to 24-hour fasting during adolescence (PN29) [10]. Nevertheless, our data show that repeated food deprivations that occurred in the ADF regimen resulted in an attenuation of this increase in corticosterone levels in the adversely reared animals fed a chow diet. However, this effect was not observed in adversely reared animals fed a cafeteria diet in the ADF regimen, suggesting that the type of diet may modulate the intensity of the stress response in adversely, but not normally, reared animals.

Our results corroborate a previous rodent study [56] showing that terminal 24-hour fasting promoted a higher increase in corticosterone levels in the ADF compared to AL regimen when normally reared animals consumed chow diet. Moreover, other studies using ADF have also reported comparable increases in corticosterone levels even when animals were not fasted [57,58]. A similar increase in cortisol levels following intermittent fasting has also been reported in clinical studies [59]. Beyond the classical roles of corticosterone in stress response, increased corticosterone levels have been implicated in energy metabolism, increased food intake and weight grain [60,61]. Thus, the increase in corticosterone levels following terminal 24-hour fasting in the animals in the ADF regimen may be involved in regulating food consumption. Indeed, a previous study using a similar ADF protocol in maternally separated animals observed compensatory food intake in refeeding days [10]. In the current study, we did not record food consumption, however, reduced body weight and fat deposition observed in the adversely reared animals fed chow diet in the ADF regimen likely reflects corticosterone-related changes in food consumption given that these animals showed a slight attenuation of corticosterone levels as compared to the ADF animals.

### Immune responses to cafeteria diet and ADF in animals exposed to early-life adversity

Exposure to childhood adversity has been shown to increase the risk for chronic health problems such as diabetes, cardiovascular diseases, cancer, and mental health problems later in life [1–7,62]. This adversity-related increase in risk for disease seems to have its roots in a chronic, low-grade pro-inflammatory bias observed following adversity during infancy [62,63]. In fact, childhood adversity is associated with increased levels of pro-inflammatory cytokines including IL-6 and TNF-α [64]. Animal models have replicated this association between early-life adversity and chronic low-grade pro-inflammatory bias later in life [12,65–68].

One of the most well-described associations between in childhood trauma and adult inflammation involves inflammatory pathways related to TNF-α [64]. We have previously demonstrated that early-life adversity induces a pro-inflammatory state, as evidenced by increased plasma levels of TNF-α and IL-6 in adversely reared animals fed chow diet [12]. Notably, these increases were observed in animals that had not been subjected to fasting. In the present study, we observed that terminal 24-hour fasting TNF-α levels were increased in adversely reared animals fed chow diet on the ADF regimen but not in those on the AL regimen. Taken together, the findings from our previous study [12] and the current results suggest that 24-hour fasting can eliminate the observed increased in TNF-α levels in adversely reared, AL-regimented animals consuming chow diet [12]. However, adversely, ADF-regimented animals fed chow diet still exhibited increased TNF-α levels, suggesting that the ADF regimen differentially affected adversely reared animals. Notably, TNF-α signaling can be impacted by other signaling molecules, including glucocorticoids, which have strong anti-inflammatory properties and are capable of inhibiting TNF-α signaling [69]. Here, we show that adversely reared animals fed chow diet on the ADF regimen had a smaller increase in terminal 24-hour fasting corticosterone levels, which may explain the correspondingly increased TNF-α levels observed in these animals.

We also replicated and expanded on our previous IL-1β findings [12] and found that adversely reared animals fed cafeteria diet had increased IL-1β levels not only in AL- as well as ADF-regimented animals. This increased IL-1β effect in adversely reared animals fed cafeteria diet is robust, having been observed in fed animals [12] as well as in fasting animals from the current study, highlighting how consumption of high-calorie, sugar/fat-enriched foods like those found in the cafeteria diet can be more detrimental to individuals exposed to early-life adversity. This differential sensitivity to diet induced by early adversity is of particular concern, as the clinical literature indicates that individuals exposed to childhood abuse are at a higher risk for emotional eating as a coping strategy to deal with abuse-related depression and/or emotional dysregulation [70].

Terminal 24-hour fasting IL-6 levels were also differentially expressed between rearing groups fed chow diet on the ADF regimen, with increased levels found in normally reared animals but not in adversely reared animals. Fasting-induced increases in IL-6 levels have also been observed in mice [71]. In humans, IL-6 has been shown to stimulate lipolysis and fat oxidation [72], and has been proposed as a possible mechanism mediating the metabolic switch from carbohydrate to lipid oxidation during fasting [71]. As such, increased IL-6 levels in normally reared, ADF-regimented animals fed chow diet may explain their observed reduction in retroperitoneal fat accumulation. However, adversely reared animals fed chow diet on the ADF regimen did not show the same increase in IL-6, suggesting an alternate mechanism for mediating the reduced perigonadal and retroperitoneal fat accumulation in these animals.

Our results also showed that adverse rearing increased terminal 24-hour fasting IL-10 levels, regardless of diet or feeding regimen. As an anti-inflammatory cytokine, IL-10 helps to dampen immune responses by inhibiting the production of pro-inflammatory cytokines, such as TNF-α, IL-1β, and IL-6. The role of IL-10 in inhibiting the production of pro-inflammatory cytokines is critical for maintaining immune system homeostasis and helping prevent immune responses from becoming excessive or prolonged [73,74]. High levels of IL-10 may be counterbalancing the inflammatory effects of pro-inflammatory cytokines, which may represent an adaptation for maintaining homeostasis in adversely reared animals.

Work in humans and animal models indicate that intermittent fasting can reduce oxidative damage and inflammation [75–77]. Our finding that ADF reduced terminal 24-hour fasting TBARS levels, a main marker of oxidative stress-induced cell damage, corroborates previous data showing that ADF can exert a beneficial antioxidant effect that reduces oxidative damage. Importantly, ADF effects were similar between normally and adversely reared animals, independent of diet, suggesting that neither early-life experiences nor diet type consumed influenced the positive effects of ADF on TBARS. However, adversely reared animals fed cafeteria diet on the AL regimen showed the highest TBARS levels. We have previously shown that adversely reared animals fed chow or cafeteria diet in the AL regimen showed decreased levels of TBARS [12]. In the current study, however, we collected fasted samples and found increased TBARS levels in adversely reared animals fed cafeteria diet on the AL regimen. Collectively, these findings highlight that early-life adversity may increase vulnerability to fasting given their higher oxidative stress response compared to controls, especially when fed cafeteria diet.

### Limitations

Given that early-life adversity [78,79], cafeteria diet [80,81], and ADF [82] are known to have sex-specific impacts, a limitation of the current study is the use of only males in the experimental design. Future studies should include both males and females to determine if the differential responses to cafeteria diet and/or ADF observed in adversely reared males are also present in females. Moreover, in the current study, the ADF regimen was employed from early adolescence to early adulthood [83], a period of intensive growth during which the organism requires a constant supply of large amounts of energy. Implementing ADF during this critical developmental period may have led to a mild form of stunting, as animals fed chow diet on the ADF regimen showed smaller body weights for their age, especially those exposed to early-life adversity. Future studies should evaluate the effects of ADF after animals have completed their growth phase to determine its impact on fully developed organisms. Nevertheless, clinical studies have evaluated the feasibility and efficacy of ADF as a possible intervention to treat obesity during adolescence [84–87]. Overall, the results indicate that ADF during adolescence can reduce BMI and cardiovascular disease risk [84,88]. However, studies highlight the need for careful consideration when using ADF during adolescence to meet the increased nutritional and energetic requirements during this critical growth period [85]. Moreover, some studies suggest caution, as adolescence is a period of increased risk for eating disorders, and ADF or any other calorie restriction intervention may further exacerbate this risk [87]. Finally, in the current study, food intake and body weight data were not collected during the entire period in which animals were fed cafeteria diet in AL or ADF regimens. However, we used the exact same early-life adversity and cafeteria diet protocols as in our previous study [12], in which we showed that animals fed cafeteria diet had a higher energy intake compared to animals fed chow diet. Importantly, adversely reared animals fed cafeteria diet displayed an even higher energy intake compared to normally reared animals fed cafeteria diet [12]. Unfortunately, we do not have data to evaluate whether the ADF regimen affected the body weight and intake of chow or cafeteria diet. However, previous research evaluating how early-life adversity (i.e., maternal separation) affects body weight and food intake using the ADF regimen demonstrated that animals exposed to early-life adversity weighed more and consumed more chow diet on fed days of the ADF regimen compared to normally reared animals [10].

## Conclusions and implications

Overall, our findings add to the literature on how exposure to early-life adversity may alter physiological responses differentially, not only to specific diets but also to consumption patterns. Indeed, adversely reared animals fed chow diet on the ADF regimen showed the largest reductions in body weight and perigonadal and retroperitoneal fat pad weight, the smallest increase in corticosterone levels, and the largest increase in TNF-α levels. However, the differential effects of the ADF regimen on body, perigonadal and retroperitoneal fat weight observed in adversely reared animals fed chow diet compared to normally reared were not present if the adversely reared animals were fed cafeteria diet on the ADF regimen. Furthermore, adversely reared animals fed cafeteria diet on the ADF regimen showed high levels of IL-1β and IL-6. Together, these data suggest that the altered vulnerability to metabolic and immune dysfunction faced by individuals exposed to early-life adversity is not just due to the type of diet but also how the diet is consumed.

## Supporting information

S1 File Data used for statistical analyses and figures. (DOCX)

## References

[1] Boynton-Jarrett R, Rosenberg L, Palmer JR, Boggs DA, Wise LA. Child and adolescent abuse in relation to obesity in adulthood: the Black women’s health study. Pediatrics. 2012;130(2):245–53. doi: 10.1542/peds.2011-1554 22753562 PMC3408680

[2] Danese A, Tan M. Childhood maltreatment and obesity: systematic review and meta-analysis. Mol Psychiatry. 2014;19(5):544–54. doi: 10.1038/mp.2013.54 23689533

[3] Flores-Torres MH, Comerford E, Signorello L, Grodstein F, Lopez-Ridaura R, de Castro F, et al. Impact of adverse childhood experiences on cardiovascular disease risk factors in adulthood among Mexican women. Child Abuse Negl. 2020;99 104175. doi: 10.1016/j.chiabu.2019.104175 31710961

[4] Gunstad J, Paul RH, Spitznagel MB, Cohen RA, Williams LM, Kohn M, et al. Exposure to early life trauma is associated with adult obesity. Psychiatry Res. 2006;142(1):31–7. doi: 10.1016/j.psychres.2005.11.007 16713630

[5] Huffhines L, Noser A, Patton SR. The link between adverse childhood experiences and diabetes. Curr Diab Rep. 2016;16(6):54. doi: 10.1007/s11892-016-0740-8 27112958 PMC5292871

[6] Lee C, Tsenkova V, Carr D. Childhood trauma and metabolic syndrome in men and women. Soc Sci Med. 2014; 105:122–30. doi: 10.1016/j.socscimed.2014.01.017 24524907 PMC4097386

[7] Rich-Edwards JW, Spiegelman D, Lividoti Hibert EN, Jun H-J, Todd TJ, Kawachi I, et al. Abuse in childhood and adolescence as a predictor of type 2 diabetes in adult women. Am J Prev Med. 2010;39(6):529–36. doi: 10.1016/j.amepre.2010.09.007 21084073 PMC3003936

[8] Bernardi JR, Ferreira CF, Senter G, Krolow R, de Aguiar BW, Portella AK, et al. Early life stress interacts with the diet deficiency of omega-3 fatty acids during the life course increasing the metabolic vulnerability in adult rats. PLoS One. 2013;8(4):e62031. doi: 10.1371/journal.pone.0062031 23614006 PMC3629088

[9] Kaufman D, Banerji MA, Shorman I, Smith ELP, Coplan JD, Rosenblum LA, et al. Early-life stress and the development of obesity and insulin resistance in juvenile bonnet macaques. Diabetes. 2007;56(5):1382–6. doi: 10.2337/db06-1409 17470564

[10] Ryu V, Lee J-H, Yoo SB, Gu XF, Moon YW, Jahng JW. Sustained hyperphagia in adolescent rats that experienced neonatal maternal separation. Int J Obes (Lond). 2008;32(9):1355–62. doi: 10.1038/ijo.2008.108 18645575

[11] Walker C-D, Bath KG, Joëls M, Korosi A, Larauche M, Lucassen PJ, et al. Chronic early life stress induced by limited bedding and nesting (LBN) material in rodents: Critical considerations of methodology, outcomes and translational potential. Stress. 2017; 20:421–48. doi: 10.1080/10253890.2017.1343296 28617197 PMC5705407

[12] Sagae SC, Zanardini B, Ribeiro-Paz ED, Amaral AC, Bronczek GA, Lubaczeuski C, et al. Metabolic dysfunction in a rat model of early-life scarcity-adversity: Modulatory role of cafeteria diet. Exp Physiol. 2018;103(11):1481–93. doi: 10.1113/EP087171 30211444

[13] Eller OC, Morris EM, Thyfault JP, Christianson JA. Early life stress reduces voluntary exercise and its prevention of diet-induced obesity and metabolic dysfunction in mice. Physiol Behav. 2020;223:113000. doi: 10.1016/j.physbeh.2020.113000 32512033 PMC7397992

[14] Jahng JW, Yoo SB, Kim JY, Kim B-T, Lee J-H. Increased mesohippocampal dopaminergic activity and improved depression-like behaviors in maternally separated rats following repeated fasting/refeeding cycles. J Obes. 2012;2012:497101. doi: 10.1155/2012/497101 22934157 PMC3425808

[15] Joslin PMN, Bell RK, Swoap SJ. Obese mice on a high-fat alternate-day fasting regimen lose weight and improve glucose tolerance. J Anim Physiol Anim Nutr (Berl). 2017;101(5):1036–45. doi: 10.1111/jpn.12546 27273295

[16] Muñoz-Hernández L, Márquez-López Z, Mehtta R, Aguiar-Salinas CA. Intermittent fasting as part of management for T2SM: from animal models to human clinical studies. Curr Diab Rep. 2020; 20:13. doi: 10.1007/s11892-020-1295-2 32166554

[17] Patterson RE, Laughlin GA, LaCroix AZ, Hartman SJ, Natarajan L, Senger CM, et al. Intermittent fasting and human metabolic health. J Acad Nutr Diet. 2015;115(8):1203–12. doi: 10.1016/j.jand.2015.02.018 25857868 PMC4516560

[18] Swoap SJ, Bingaman MJ, Hult EM, Sandstrom NJ. Alternate-day feeding leads to improved glucose regulation on fasting days without significant weight loss in genetically obese mice. Am J Physiol Regul Integr Comp Physiol. 2019;317:R461–9. doi: 10.1152/ajpregu.00140.2019 31290685

[19] Paternain L, Martisova E, Milagro FI, Ramírez MJ, Martínez JA, Campión J. Postnatal maternal separation modifies the response to an obesogenic diet in adulthood in rats. Dis Model Mech. 2012;5(5):691–7. doi: 10.1242/dmm.009043 22773756 PMC3424467

[20] Ruigrok SR, Abbink MR, Geertsema J, Kuindersma JE, Stöberl N, van der Beek EM, et al. Effects of early-life stress, postnatal diet modulation and long-term western-style diet on peripheral and central inflammatory markers. Nutrients. 2021;13(2):288. doi: 10.3390/nu13020288 33498469 PMC7909521

[21] Yam KY, Naninck EFG, Abbink MR, la Fleur SE, Schipper L, van den Beukel JC, et al. Exposure to chronic early-life stress lastingly alters the adipose tissue, the leptin system and changes the vulnerability to western-style diet later in life in mice. Psychoneuroendocrinology. 2017;77:186–95. doi: 10.1016/j.psyneuen.2016.12.012 28088658

[22] Raineki C, Opendak M, Sarro E, Showler A, Bui K, McEwen BS, et al. During infant maltreatment, stress targets hippocampus, but stress with mother present targets amygdala and social behavior. Proc Natl Acad Sci U S A. 2019;116(45):22821–32. doi: 10.1073/pnas.1907170116 31636210 PMC6842629

[23] Raineki C, Sarro E, Rincón-Cortés M, Perry R, Boggs J, Holman CJ, et al. Paradoxical neurobehavioral rescue by memories of early-life abuse: the safety signal value of odors learned during abusive attachment. Neuropsychopharmacology. 2015;40(4):906–14. doi: 10.1038/npp.2014.266 25284320 PMC4330504

[24] Sagae SC, Menezes EF, Bonfleur ML, Vanzela EC, Zacharias P, Lubaczeuski C, et al. Early onset of obesity induces reproductive deficits in female rats. Physiol Behav. 2012;105(5):1104–11. doi: 10.1016/j.physbeh.2011.12.002 22178647

[25] Sagae SC, Lubaczeuski C, Zacharias P, Bonfleur ML, Franci CR, Sanvitto GL, et al. Prevention of metabolic disorders and reproductive performance deficits by the blockade of angiotensin II AT1 receptor in female rats fed with cafeteria diet. Physiol Behav. 2013;119:1–8. doi: 10.1016/j.physbeh.2013.05.029 23727535

[26] Kurosawa S, Kato M. Anesthetics, immune cells, and immune responses. J Anesth. 2008;22(3):263–77. doi: 10.1007/s00540-008-0626-2 18685933

[27] Malcon LMC, Wearick-Silva LE, Zaparte A, Orso R, Luft C, Tractenberg SG, et al. Maternal separation induces long-term oxidative stress alterations and increases anxiety-like behavior of male Balb/cJ mice. Exp Brain Res. 2020;238(9):2097–107. doi: 10.1007/s00221-020-05859-y 32656651

[28] Gomes-Smith M, Karthikeyan S, Jeffers M, Janik R, Thomason L, Stefanovic B. A physiological characterization of the cafeteria diet model of metabolic syndrome in the rat. Physiol Behav. 2016;167 382–91. doi: 10.1016/j.physbeh.2016.09.029 27705750

[29] Sampey BP, Vanhoose AM, Winfield HM, Freemerman AJ, Muehlbauer MJ, Fueger PT, et al. Cafeteria diet is a robust model of human metabolic syndrome with liver and adipose inflammation: comparison to high-fat diet. Obesity (Silver Spring). 2011;19(6):1109–17. doi: 10.1038/oby.2011.18 21331068 PMC3130193

[30] Lalanza J, Snoeren E. The cafeteria diet: a standardized protocol and its effects on behavior. Neurosci Biobehav Rev. 2021;122:92–119. doi: 10.1016/j.neubiorev.2020.11.003 33309818

[31] de Schepper J, Zhou X, Louis O, Velkeniers B, Hooghe-Peters E. The weight gain and ultimate adiposity in cafeteria diet-induced obesity is unrelated to the central serotoninergic tonus. Eating Weight Disord. 1997;1:38–43. doi: 10.1007/BF03339948 14655855

[32] Ferreira A, Castro JP, Andrade JP, Dulce Madeira M, Cardoso A. Cafeteria-diet effects on cognitive functions, anxiety, fear response and neurogenesis in the juvenile rat. Neurobiol Learn Mem. 2018;155:197–207. doi: 10.1016/j.nlm.2018.07.014 30075193

[33] Kitchell BB. Heart and liver lipid fatty acid and behavior changes in mice after a diet change. Life Sci. 1984;34(17):1613–20. doi: 10.1016/0024-3205(84)90631-3 6727538

[34] Kosheleff AR, Araki J, Tsan L, Chen G, Murphy NP, Maidment NT, et al. Junk food exposure disrupts selection of food-seeking actions in rats. Front Psychiatry. 2018;9:350. doi: 10.3389/fpsyt.2018.00350 30166974 PMC6106797

[35] Pérez C, Fanizza LJ, Sclafani A. Flavor preferences conditioned by intragastric nutrient infusions in rats fed chow or a cafeteria diet. Appetite. 1999;32(1):155–70. doi: 10.1006/appe.1998.0182 9989925

[36] Pini RTB, do Vales LDMF, Costa TMB, Almeida SS. Effects of cafeteria diet and high fat diet intake on anxiety, learning and memory in adult male rats. Nutr Neurosci. 2017;20:396–408. doi: 10.1080/1028415X.2016.114929 28277186

[37] Warneke W, Klaus S, Fink H, Langley-Evans SC, Voigt J-P. The impact of cafeteria diet feeding on physiology and anxiety-related behaviour in male and female Sprague-Dawley rats of different ages. Pharmacol Biochem Behav. 2014;116 45–54. doi: 10.1016/j.pbb.2013.11.016 24269545

[38] Archer ZA, Rayner DV, Mercer JG. Hypothalamic gene expression is altered in underweight but obese juvenile male Sprague-Dawley rats fed a high-energy diet. J Nutr. 2004;134(6):1369–74. doi: 10.1093/jn/134.6.1369 15173398

[39] Lettieri-Barbato D, Giovannetti E, Aquilano K. Effects of dietary restriction on adipose mass and biomarkers of healthy aging in human. Aging (Albany NY). 2016;8(12):3341–55. doi: 10.18632/aging.101122 27899768 PMC5270672

[40] Patterson RE, Sears DD. Metabolic effects of intermittent fasting. Annu Rev Nutr. 2017;37:371–93. doi: 10.1146/annurev-nutr-071816-064634 28715993 PMC13170603

[41] Kochan Z, Goyke E, Karbowska J, Slominska E, Swierczynski J. The decrease of rat postprandial plasma triacylglycerol concentration after multiple cycles of starvation-refeeding. Horm Metab Res. 2001;33(1):26–9. doi: 10.1055/s-2001-12622 11280711

[42] Mozeš Š, Šefčíková Z, Raček Ľ. Effect of repeated fasting/refeeding on obesity development and health complications in rats arising from reduced nest. Dig Dis Sci. 2015;60(2):354–61. doi: 10.1007/s10620-014-3340-y 25150705

[43] Wilson RA, Deasy W, Stathis CG, Hayes A, Cooke MB. Intermittent fasting with or without exercise prevents weight gain and improves lipids in diet-induced obese mice. Nutrients. 2018;10(3):346. doi: 10.3390/nu10030346 29534545 PMC5872764

[44] Soeters MR, Soeters PB, Schooneman MG, Houten SM, Romijn JA. Adaptive reciprocity of lipid and glucose metabolism in human short-term starvation. Am J Physiol Endocrinol Metab. 2012;303(12):E1397–407. doi: 10.1152/ajpendo.00397.2012 23074240

[45] Desautels M, Dulos RA. Effects of repeated cycles of fasting-refeeding on brown adipose tissue composition in mice. Am J Physiol. 1988;255(2 Pt 1):E120–8. doi: 10.1152/ajpendo.1988.255.2.E120 3407768

[46] Townsend KL, Tseng Y-H. Brown fat fuel utilization and thermogenesis. Trends Endocrinol Metab. 2014;25(4):168–77. doi: 10.1016/j.tem.2013.12.004 24389130 PMC3972344

[47] Chondronikola M, Volpi E, Børsheim E, Porter C, Saraf MK, Annamalai P, et al. Brown adipose tissue activation is linked to distinct systemic effects on lipid metabolism in humans. Cell Metab. 2016;23(6):1200–6. doi: 10.1016/j.cmet.2016.04.029 27238638 PMC4967557

[48] Matsushita M, Yoneshiro T, Aita S, Kameya T, Sugie H, Saito M. Impact of brown adipose tissue on body fatness and glucose metabolism in healthy humans. Int J Obes (Lond). 2014;38(6):812–7. doi: 10.1038/ijo.2013.206 24213309

[49] Vegiopoulos A, Müller-Decker K, Strzoda D, Schmitt I, Chichelnitskiy E, Ostertag A, et al. Cyclooxygenase-2 controls energy homeostasis in mice by de novo recruitment of brown adipocytes. Science. 2010;328(5982):1158–61. doi: 10.1126/science.1186034 20448152

[50] Glick Z, Teague RJ, Bray GA. Brown adipose tissue: thermic response increased by a single low protein, high carbohydrate meal. Science. 1981;213:1125–7. doi: 10.1126/science.7268419 7268419

[51] Lupien JR, Glick Z, Saito M, Bray GA. Guanosine diphosphate binding to brown adipose tissue mitochondria is increased after single meal. Am J Physiol. 1985;249:R694–8. doi: 10.1152/ajpregu.1985.249.6.R694 3000200

[52] Chaves VE, Frasson D, Martins-Santos MES, Navegantes LCC, Galban VD, Garófalo MAR, et al. Fatty acid synthesis and generation of glycerol-3-phosphate in brown adipose tissue from rats fed a cafeteria diet. Can J Physiol Pharmacol. 2008;86(7):416–23. doi: 10.1139/y08-052 18641690

[53] Liu H, Javaheri A, Godar RJ, Murphy J, Ma X, Rohatgi N, et al. Intermittent fasting preserves beta-cell mass in obesity-induced diabetes via the autophagy-lysosome pathway. Autophagy. 2017;13(11):1952–68. doi: 10.1080/15548627.2017.1368596 28853981 PMC5788488

[54] Kang S, Ahn E, Cha Y-C. Changes in lipid and carnitine concentrations following repeated fasting-refeeding in mice. Nutr Res Pract. n2010;4:477–85. doi: 10.4162/nrp.2010.4.6.477 21286405 PMC3029788

[55] Kale VP, Joshi GS, Gohil PB, Jain MR. Effect of fasting duration on clinical pathology results in Wistar rats. Vet Clin Pathol. 2009;38(3):361–6. doi: 10.1111/j.1939-165X.2009.00143.x 19351329

[56] Cignarella F, Cantoni C, Ghezzi L, Salter A, Dorsett Y, Chen L, et al. Intermittent fasting confers protection in CNS autoimmunity by altering the gut microbiota. Cell Metab. 2018;27(6):1222–35.e6. doi: 10.1016/j.cmet.2018.05.006 29874567 PMC6460288

[57] Wan R, Camandola S, Mattson MP. Intermittent fasting and dietary supplementation with 2-deoxy-D-glucose improve functional and metabolic cardiovascular risk factors in rats. FASEB J. 2003;17(9):1133–4. doi: 10.1096/fj.02-0996fje 12709404

[58] Zenz G, Jačan A, Reichmann F, Farzi A, Holzer P. Intermittent fasting exacerbates the acute immune and behavioral sickness response to the viral mimic poly(I:C) in mice. Front Neurosci. 2019;13:359. doi: 10.3389/fnins.2019.00359 31057355 PMC6478699

[59] Kim B, Joo Y, Kim M-S, Choe H, Tong Q, et al. Effects of intermittent fasting on the circulating levels and circadian rhythms of hormones. Endocrinol Metab. 2021;36:745–56. 10.3803/EnM.2021.405 PMC8419605 34474513

[60] Dallman MF. Stress-induced obesity and the emotional nervous system. Trends Endocrinol Metab. 2010;21(3):159–65. doi: 10.1016/j.tem.2009.10.004 19926299 PMC2831158

[61] Sapolsky RM, Romero LM, Munck AU. How do glucocorticoids influence stress responses? Integrating permissive, suppressive, stimulatory, and preparative actions. Endocr Rev. 2000;21(1):55–89. doi: 10.1210/edrv.21.1.0389 10696570

[62] Miller GE, Chen E, Parker KJ. Psychological stress in childhood and susceptibility to the chronic diseases of aging: moving toward a model of behavioral and biological mechanisms. Psychol Bull. 2011;137(6):959–97. doi: 10.1037/a0024768 21787044 PMC3202072

[63] Nathan C, Ding A. Nonresolving inflammation. Cell. 2010;140(6):871–82. doi: 10.1016/j.cell.2010.02.029 20303877

[64] Coelho R, Viola TW, Walss-Bass C, Brietzke E, Grassi-Oliveira R. Childhood maltreatment and inflammatory markers: a systematic review. Acta Psychiatr Scand. 2014;129(3):180–92. doi: 10.1111/acps.12217 24205846

[65] Ganguly P, Brenhouse HC. Broken or maladaptive? Altered trajectories in neuroinflammation and behavior after early life adversity. Dev Cogn Neurosci. 2015;11:18–30. doi: 10.1016/j.dcn.2014.07.001 25081071 PMC4476268

[66] Pinheiro RMC, de Lima MNM, Portal BCD, Busato SB, Falavigna L, Ferreira RDP, et al. Long-lasting recognition memory impairment and alterations in brain levels of cytokines and BDNF induced by maternal deprivation: effects of valproic acid and topiramate. J Neural Transm (Vienna). 2015;122(5):709–19. doi: 10.1007/s00702-014-1303-2 25182413

[67] Raineki C, Bodnar TS, Holman PJ, Baglot SL, Lan N, Weinberg J. Effects of early-life adversity on immune function are mediated by prenatal environment: Role of prenatal alcohol exposure. Brain Behav Immun. 2017;66:210–20. doi: 10.1016/j.bbi.2017.07.001 28698116 PMC5650917

[68] Réus GZ, Dos Santos MAB, Abelaira HM, Ribeiro KF, Petronilho F, Vuolo F, et al. Imipramine reverses alterations in cytokines and BDNF levels induced by maternal deprivation in adult rats. Behav Brain Res. 2013;242:40–6. doi: 10.1016/j.bbr.2012.11.044 23238043

[69] Van Bogaert T, De Bosscher K, Libert C. Crosstalk between TNF and glucocorticoid receptor signaling pathways. Cytokine Growth Factor Rev. 2010;21(4):275–86. doi: 10.1016/j.cytogfr.2010.04.003 20456998

[70] Michopoulos V, Powers A, Moore C, Villarreal S, Ressler KJ, Bradley B, et al. The mediating role of emotion dysregulation and depression on the relationship between childhood trauma exposure and emotional eating. Appetite. 2015;91:129–36. doi: 10.1016/j.appet.2015.03.036 25865667 PMC4459910

[71] Wueest S, Item F, Boyle CN, Jirkof P, Cesarovic N, Ellingsgaard H, et al. Interleukin-6 contributes to early fasting-induced free fatty acid mobilization in mice. Am J Physiol Regul Integr Comp Physiol. 2014;306(11):R861–7. doi: 10.1152/ajpregu.00533.2013 24694381

[72] van Hall G, Steensberg A, Sacchetti M, Fischer C, Keller C, Schjerling P, et al. Interleukin-6 stimulates lipolysis and fat oxidation in humans. J Clin Endocrinol Metab. 2003;88(7):3005–10. doi: 10.1210/jc.2002-021687 12843134

[73] Jaffer U, Wade RG, Gourlay T. Cytokines in the systemic inflammatory response syndrome: a review. HSR Proc Intensive Care Cardiovasc Anesth. 2010;2(3):161–75. 23441054 PMC3484588

[74] Shouval DS, Biswas A, Goettel JA, McCann K, Conaway E, Redhu NS, et al. Interleukin-10 receptor signaling in innate immune cells regulates mucosal immune tolerance and anti-inflammatory macrophage function. Immunity. 2014; 40:706–19. 24792912 10.1016/j.immuni.2014.03.011 PMC4513358

[75] Castello L, Froio T, Maina M, Cavallini G, Biasi F, Leonarduzzi G, et al. Alternate-day fasting protects the rat heart against age-induced inflammation and fibrosis by inhibiting oxidative damage and NF-kB activation. Free Radic Biol Med. 2010;48(1):47–54. doi: 10.1016/j.freeradbiomed.2009.10.003 19818847

[76] Descamps O, Riondel J, Ducros V, Roussel A-M. Mitochondrial production of reactive oxygen species and incidence of age-associated lymphoma in OF1 mice: effect of alternate-day fasting. Mech Ageing Dev. 2005;126(11):1185–91. doi: 10.1016/j.mad.2005.06.007 16126250

[77] Johnson JB, Summer W, Cutler RG, Martin B, Hyun D-H, Dixit VD, et al. Alternate day calorie restriction improves clinical findings and reduces markers of oxidative stress and inflammation in overweight adults with moderate asthma. Free Radic Biol Med. 2007;42(5):665–74. doi: 10.1016/j.freeradbiomed.2006.12.005 17291990 PMC1859864

[78] Goodwill HL, Manzano-Nieves G, Gallo M, Lee H-I, Oyerinde E, Serre T, et al. Early life stress leads to sex differences in development of depressive-like outcomes in a mouse model. Neuropsychopharmacology. 2019;44(4):711–20. doi: 10.1038/s41386-018-0195-5 30188513 PMC6372611

[79] Grassi-Oliveira R, Honeycutt JA, Holland FH, Ganguly P, Brenhouse HC. Cognitive impairment effects of early life stress in adolescents can be predicted with early biomarkers: Impacts of sex, experience, and cytokines. Psychoneuroendocrinology. 2016;71:19–30. doi: 10.1016/j.psyneuen.2016.04.016 27235636 PMC5412140

[80] Gual-Grau A, Guirro M, Boqué N, Arola L. Physiological, metabolic and microbial responses to obesogenic cafeteria diet in rats: The impact of strain and sex. J Nutr Biochem. 2023;117:109338. doi: 10.1016/j.jnutbio.2023.109338 36997035

[81] Ong ZY, Wanasuria AF, Lin MZP, Hiscock J, Muhlhausler BS. Chronic intake of a cafeteria diet and subsequent abstinence. sex-specific effects on gene expression in the mesolimbic reward system. Appetite. 2013;65:189–99. doi: 10.1016/j.appet.2013.01.014 23402719

[82] Piotrowska K, Tarnowski M, Zgutka K, Pawlik A. Gender differences in response to prolonged every-other-day feeding on the proliferation and apoptosis of hepatocytes in mice. Nutrients. 2016;8(3):176. doi: 10.3390/nu8030176 27007393 PMC4808902

[83] Holder MK, Blaustein JD. Puberty and adolescence as a time of vulnerability to stressors that alter neurobehavioral processes. Front Neuroendocrinol. 2014;35(1):89–110. doi: 10.1016/j.yfrne.2013.10.004 24184692 PMC3946873

[84] Jebeile H, Gow ML, Lister NB, Mosalman Haghighi M, Ayer J, Cowell CT, et al. Intermittent energy restriction is a feasible, effective, and acceptable intervention to treat adolescents with obesity. J Nutr. 2019;149(7):1189–97. doi: 10.1093/jn/nxz049 31006807

[85] Lister NB, Gow ML, Chisholm K, Grunseit A, Garnett SP, Baur LA. Nutritional adequacy of diets for adolescents with overweight and obesity: considerations for dietetic practice. Eur J Clin Nutr. 2017;71(5):646–51. doi: 10.1038/ejcn.2016.268 28225054

[86] Lister NB, Jebeile H, Truby H, Garnett SP, Varady KA, Cowell CT, et al. Fast track to health - Intermittent energy restriction in adolescents with obesity. a randomised controlled trial study protocol. Obes Res Clin Pract. 2020;14(1):80–90. doi: 10.1016/j.orcp.2019.11.005 31818675

[87] Varady KA, Cienfuegos S, Ezpeleta M, Gabel K. Clinical application of intermittent fasting for weight loss: progress and future directions. Nat Rev Endocrinol. 2022;18(5):309–21. doi: 10.1038/s41574-022-00638-x 35194176

[88] Lister N, House R, Kwok C, Inkster M, Day K. A 12-month randomized controlled trial using intensive dietary interventions for adolescents with obesity associated with complications. Proc Nutr Soc. 2024;83:E172.

