# Supplementary material for: Alternate-day fasting differentially affects body composition, metabolic and immune response to fasting in male rats exposed to early-life adversity: Modulatory role of cafeteria diet
Source: PLoS One. 2025 Mar 3;20(3):e0313103. doi: 10.1371/journal.pone.0313103 (PMC11875342; doi:10.1371/journal.pone.0313103)
Supplement: S1 File — (DOCX) [file pone.0313103.s001.docx]

**Supplementary Table 1.** Number of animals in each experimental group

|  | Normal rearing | | | | Adverse rearing | | | |
| --- | --- | --- | --- | --- | --- | --- | --- | --- |
|  | Chow diet | | Cafeteria diet | | Chow diet | | Cafeteria diet | |
|  | AL | ADF | AL | ADF | AL | ADF | AL | ADF |
| Body weight | 5 | 6 | 5 | 6 | 4 | 6 | 4 | 6 |
| Perigonadal fat | 5 | 6 | 5 | 6 | 4 | 5 | 4 | 6 |
| Retroperitoneal fat | 5 | 6 | 5 | 6 | 4 | 6 | 4 | 6 |
| Brown fat | 9 | 6 | 6 | 6 | 9 | 6 | 7 | 5 |
| Cholesterol | 9 | 7 | 5 | 6 | 4 | 6 | 6 | 5 |
| Triglycerides | 9 | 5 | 5 | 6 | 4 | 6 | 6 | 6 |
| Corticosterone | 7 | 6 | 5 | 6 | 4 | 6 | 6 | 6 |
| TNF-α | 9 | 6 | 5 | 6 | 4 | 6 | 6 | 6 |
| IL-1β | 9 | 6 | 5 | 6 | 4 | 6 | 6 | 6 |
| IL-10 | 9 | 6 | 5 | 6 | 4 | 6 | 6 | 6 |
| IL-6 | 9 | 6 | 5 | 6 | 4 | 6 | 5 | 6 |
| TBARS | 9 | 6 | 5 | 6 | 4 | 6 | 6 | 6 |

**Supplementary Table 2.** Statistical report for graphs in Figure 2

| **Outcome** | **Factors** | **F value** | **P value** |
| --- | --- | --- | --- |
| Body weight | rearing condition | F_(1,34)_ = 0.14 | 0.71 |
|  | diet | F_(1,34)_ = 1.84 | 0.18 |
|  | feeding regimen | F_(1,34)_ = 15.46 | **0.0003** |
|  | rearing condition and diet | F_(1,34)_ = 0.05 | 0.83 |
|  | rearing condition and feeding regimen | F_(1,34)_ = 4.70 | **0.04** |
|  | diet and feeding regimen | F_(1,34)_ = 1.87 | 0.18 |
|  | rearing condition, diet and feeding regimen | F_(1,34)_ = 0.81 | 0.37 |
| Perigonadal fat | rearing condition | F_(1,33)_ = 3.66 | 0.06 |
|  | diet | F_(1,33)_ = 37.03 | **0.0001** |
|  | feeding regimen | F_(1,33)_ = 42.41 | **0.0001** |
|  | rearing condition and diet | F_(1,33)_ = 2.55 | 0.12 |
|  | rearing condition and feeding regimen | F_(1,33)_ = 0.52 | 0.48 |
|  | diet and feeding regimen | F_(1,33)_ = 1.89 | 0.18 |
|  | rearing condition, diet and feeding regimen | F_(1,33)_ = 6.29 | **0.02** |
| Retroperitoneal fat | rearing condition | F_(1,34)_ = 2.25 | 0.14 |
|  | diet | F_(1,34)_ = 157.55 | **0.0001** |
|  | feeding regimen | F_(1,34)_ = 138.05 | **0.0001** |
|  | rearing condition and diet | F_(1,34)_ = 0.20 | 0.66 |
|  | rearing condition and feeding regimen | F_(1,34)_ = 0.06 | 0.82 |
|  | diet and feeding regimen | F_(1,34)_ = 15.53 | **0.0004** |
|  | rearing condition, diet and feeding regimen | F_(1,34)_ = 0.30 | 0.59 |
| Brown fat | rearing condition | F_(1,46)_ = 1.29 | 0.26 |
|  | diet | F_(1,46)_ = 32.67 | **0.0001** |
|  | feeding regimen | F_(1,46)_ = 12.62 | **0.0009** |
|  | rearing condition and diet | F_(1,46)_ = 0.12 | 0.73 |
|  | rearing condition and feeding regimen | F_(1,46)_ = 0.51 | 0.48 |
|  | diet and feeding regimen | F_(1,46)_ = 12.37 | **0.0009** |
|  | rearing condition, diet and feeding regimen | F_(1,46)_ = 0.24 | 0.62 |

**Supplementary Table 3.** Statistical report for graphs in Figure 3

| **Outcome** | **Factors** | **F value** | **P value** |
| --- | --- | --- | --- |
| Cholesterol | rearing condition | F_(1,40)_ = 6.59 | **0.01** |
|  | diet | F_(1,40)_ = 16.36 | **0.0002** |
|  | feeding regimen | F_(1,40)_ = 37.09 | **0.0001** |
|  | rearing condition and diet | F_(1,40)_ = 2.71 | 0.11 |
|  | rearing condition and feeding regimen | F_(1,40)_ = 35.30 | **0.0001** |
|  | diet and feeding regimen | F_(1,40)_ = 22.24 | **0.0001** |
|  | rearing condition, diet and feeding regimen | F_(1,40)_ = 0.25 | 0.62 |
| Triglycerides | rearing condition | F_(1,40)_ = 0.05 | 0.83 |
|  | diet | F_(1,40)_ = 0.002 | 0.96 |
|  | feeding regimen | F_(1,40)_ = 4.44 | **0.04** |
|  | rearing condition and diet | F_(1,40)_ = 1.50 | 0.23 |
|  | rearing condition and feeding regimen | F_(1,40)_ = 2.75 | 0.10 |
|  | diet and feeding regimen | F_(1,40)_ = 1.74 | 0.19 |
|  | rearing condition, diet and feeding regimen | F_(1,40)_ = 1.11 | 0.30 |
| Corticosterone | rearing condition | F_(1,38)_ = 9.03 | **0.005** |
|  | diet | F_(1,38)_ = 0.77 | 0.38 |
|  | feeding regimen | F_(1,38)_ = 3.83 | 0.057 |
|  | rearing condition and diet | F_(1,38)_ = 0.04 | 0.83 |
|  | rearing condition and feeding regimen | F_(1,38)_ = 14.23 | **0.0005** |
|  | diet and feeding regimen | F_(1,38)_ = 0.03 | 0.85 |
|  | rearing condition, diet and feeding regimen | F_(1,38)_ = 4.99 | **0.03** |

**Supplementary Table 4.** Statistical report for graphs in Figure 4

| **Outcome** | **Factors** | **F value** | **P value** |
| --- | --- | --- | --- |
| TNF-α | rearing condition | F_(1,40)_ = 4.61 | **0.04** |
|  | diet | F_(1,40)_ = 2.72 | 0.11 |
|  | feeding regimen | F_(1,40)_ = 1.36 | 0.25 |
|  | rearing condition and diet | F_(1,40)_ = 0.06 | 0.80 |
|  | rearing condition and feeding regimen | F_(1,40)_ = 0.16 | 0.69 |
|  | diet and feeding regimen | F_(1,40)_ = 5.09 | **0.03** |
|  | rearing condition, diet and feeding regimen | F_(1,40)_ = 4.15 | **0.05** |
| IL-1β | rearing condition | F_(1,40)_ = 7.73 | **0.008** |
|  | diet | F_(1,40)_ = 8.76 | **0.005** |
|  | feeding regimen | F_(1,40)_ = 0.25 | 0.62 |
|  | rearing condition and diet | F_(1,40)_ = 5.75 | **0.02** |
|  | rearing condition and feeding regimen | F_(1,40)_ = 0.37 | 0.55 |
|  | diet and feeding regimen | F_(1,40)_ = 0.0004 | 0.98 |
|  | rearing condition, diet and feeding regimen | F_(1,40)_ = 2.43 | 0.13 |
| IL-10 | rearing condition | F_(1,40)_ = 17.88 | **0.0001** |
|  | diet | F_(1,40)_ = 0.19 | 0.67 |
|  | feeding regimen | F_(1,40)_ = 1.09 | 0.30 |
|  | rearing condition and diet | F_(1,40)_ = 0.89 | 0.35 |
|  | rearing condition and feeding regimen | F_(1,40)_ = 1.01 | 0.32 |
|  | diet and feeding regimen | F_(1,40)_ = 0.002 | 0.96 |
|  | rearing condition, diet and feeding regimen | F_(1,40)_ = 0.89 | 0.35 |
| IL-6 | rearing condition | F_(1,39)_ = 0.21 | 0.65 |
|  | diet | F_(1,39)_ = 1.45 | 0.24 |
|  | feeding regimen | F_(1,39)_ = 0.49 | 0.49 |
|  | rearing condition and diet | F_(1,39)_ = 0.27 | 0.61 |
|  | rearing condition and feeding regimen | F_(1,39)_ = 0.37 | 0.55 |
|  | diet and feeding regimen | F_(1,39)_ = 2.46 | 0.12 |
|  | rearing condition, diet and feeding regimen | F_(1,39)_ = 7.07 | **0.01** |
| TBARS | rearing condition | F_(1,40)_ = 2.44 | 0.12 |
|  | diet | F_(1,40)_ = 2.52 | 0.12 |
|  | feeding regimen | F_(1,40)_ = 44.43 | **0.0001** |
|  | rearing condition and diet | F_(1,40)_ = 3.05 | 0.09 |
|  | rearing condition and feeding regimen | F_(1,40)_ = 10.02 | **0.003** |
|  | diet and feeding regimen | F_(1,40)_ = 1.24 | 0.27 |
|  | rearing condition, diet and feeding regimen | F_(1,40)_ = 3.66 | 0.06 |

**Supplementary Table 5.** Data expressed as mean±SEM

|  | Normal rearing | | | | Adverse rearing | | | |
| --- | --- | --- | --- | --- | --- | --- | --- | --- |
|  | Chow diet | | Cafeteria diet | | Chow diet | | Cafeteria diet | |
|  | AL | ADF | AL | ADF | AL | ADF | AL | ADF |
| Body weight | 272.2±11.46 | 258.2±4.88 | 262.0±15.46 | 253.8±8.13 | 290.5±4.99 | 237.8±5.07 | 266.3±10.09 | 242.2±6.70 |
| Perigonadal fat | 0.678±0.036 | 0.580±0.030 | 1.272±0.110 | 0.775±0.028 | 0.827±0.082 | 0.398±0.028 | 1.000±0.123 | 0.687±0.095 |
| Retroperitoneal fat | 0.732±0.064 | 0.325±0.028 | 1.694±0.101 | 0.767±0.075 | 0.665±0.122 | 0.167±0.016 | 1.615±0.159 | 0.723±0.081 |
| Brown fat | 0.073±0.007 | 0.113±0.009 | 0.123±0.010 | 0.130±0.004 | 0.070±0.005 | 0.108±0.009 | 0.122±0.008 | 0.115±0.007 |
| Cholesterol | 114.2±5.51 | 60.4±2.24 | 108.2±7.36 | 83.3±3.79 | 82.2±6.03 | 64.5±2.14 | 83.8±2.89 | 100.6±3.59 |
| Triglycerides | 75.6±3.85 | 98.6±5.30 | 64.2±9.57 | 107.8±17.83 | 70.5±7.96 | 105.7±22.21 | 116.0±23.97 | 92.0±10.55 |
| Corticosterone | 123.1±20.79 | 220.1±9.51 | 159.9±10.90 | 199.2±21.28 | 227.8±20.60 | 181.9±14.63 | 216.5±16.60 | 219.2±11.18 |
| TNF-α | 2.954±0.048 | 3.048±0.137 | 2.842±0.252 | 2.894±0.128 | 2.951±0.143 | 3.510±0.084 | 3.179±0.180 | 2.919±0.092 |
| IL-1β | 32.55±1.51 | 31.33±1.14 | 29.78±0.49 | 37.28±3.95 | 30.94±0.74 | 35.11±2.20 | 50.58±8.62 | 45.81±4.55 |
| IL-10 | 89.45±3.28 | 98.02±4.40 | 88.05±2.17 | 91.42±2.90 | 104.70±3.27 | 101.90±3.63 | 103.30±6.78 | 106.30±3.42 |
| IL-6 | 21.22±2.45 | 32.61±5.71 | 27.82±5.43 | 16.95±1.12 | 26.48±5.34 | 27.02±1.78 | 21.81±2.65 | 28.09±3.69 |
| TBARS | 3.689±0.352 | 2.400±0.139 | 3.060±0.287 | 2.967±0.198 | 3.900±0.303 | 2.117±0.138 | 4.717±0.260 | 2.617±0.248 |
